# Large schwannoma of the femur – a common tumor at an unusual site: a case report and review of the literature

**DOI:** 10.1186/s13256-017-1314-3

**Published:** 2017-05-31

**Authors:** Niranthi Perera, Chandu de Silva, Vasantha Perera

**Affiliations:** 1 Department of Pathology, Faculty of Medicine, University of Colombo, Colombo, Sri Lanka; 2 Nawaloka Hospitals PLC, Colombo, Sri Lanka

**Keywords:** Case reports, Schwannoma, Femur, Neurilemmoma

## Abstract

**Background:**

Schwannomas are benign nerve sheath tumors and are most frequently encountered as soft tissue tumors of peripheral nerves of the head and neck or the extensor extremities. Osseous involvement is very uncommon with fewer than 200 cases described in the world literature, the majority of which arise in the skull (including mandible), vertebrae, and sacrum. Long bone involvement is highly unusual and of the approximately 20 cases described thus far, only five have been documented to arise in the femur. We describe an unusually large schwannoma of the femur which was discovered incidentally and was diagnosed only after biopsy, given the rarity of this tumor at that particular site. Following prophylactic internal fixation and bone grafting, our patient remains well and disease-free, 2.5 years later.

**Case presentation:**

A 56-year-old Sri Lankan woman was discovered to have a large lytic lesion in her lower femur on routine X-ray following a fall. A history and physical examination, along with selective imaging and tissue sampling, were necessary to arrive at the diagnosis of schwannoma of the femur. The clinical presentation, radiology, pathology, and surgical management are discussed and contrasted with the other five cases documented in the literature. The tumor was successfully treated with evacuation through a lateral surgical approach and internal fixation. She remains well and disease-free 2.5 years later.

**Conclusions:**

We present the case of an unusually large lytic lesion found incidentally in the femur of a 56-year-old woman, which was subsequently diagnosed to be a schwannoma on biopsy. Its exceptional rarity in long bones makes it less likely to be considered in an initial differential diagnosis, and we stress the importance of tissue biopsy for diagnosis.

## Background

Schwannomas are benign nerve sheath tumors originating from Schwann cells and are most frequently encountered as soft tissue tumors of peripheral nerves of the head and neck or the extensor extremities [1]. Osseous involvement, however, is very uncommon, accounting for <0.2% of primary bone tumors, and when it does occur, is usually found in the mandible, spine, or sacrum [2, 3]. Schwannomas occurring in long bones are exceptionally rare and we identified only five published cases that described schwannoma in the femur [4–7] in the world literature. We present a case of an unusually large schwannoma of the lower femur, discovered incidentally on an X-ray taken in a 56-year-old Sri Lankan woman, following a fall. The clinical presentation, radiology, and pathology of the current tumor is discussed. The clinicoradiologic features of the five reported cases are compared with the current tumor. Following prophylactic internal fixation and bone grafting, the patient remains well and disease-free, 2.5 years later.

## Case presentation

A 56-year-old Sri Lankan woman presented with pain in her left knee and difficulty in weight bearing following a fall 6 weeks prior. Clinical examination of her knee and lower end of her thigh did not reveal any abnormality. She had no family history or symptoms of neurofibromatosis.

**Fig. 1:**
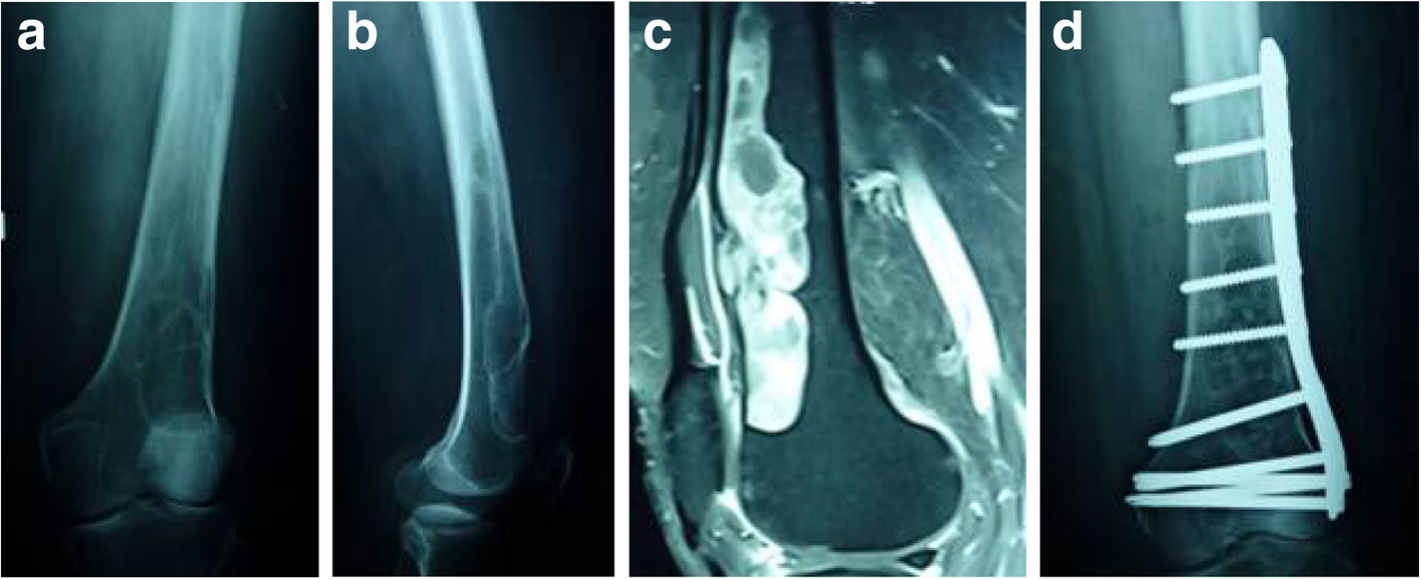
**a** Anteroposterior and **b** lateral radiographs of the distal femur showing the osteolytic lesion in the femoral cortex. **c** T1-weighted magnetic resonance imaging shows lobulated tumor. **d** Radiograph shows prophylactic internal fixation with a distal femoral plate after evacuation of tumor

Anteroposterior (Fig. 1a) and lateral (Fig. 1b) radiographs of her knee demonstrated an osteolytic lesion involving the femoral cortex, extending from 2.5 cm above the lower end of her femur to 15 cm on the lateral view. On the anteroposterior view, the lesion was in relation to the lateral cortex of her femur measuring approximately 15 cm in length and 5 cm in width. The margins of the lesion were regular with a distinct sclerotic rim, favoring a probably benign bone tumor. The anterior cortex was expanded but there was no radiologically visible extension into the adjacent soft tissue. A magnetic resonance imaging (MRI) scan (Fig. 1c) demonstrated a well-defined lobulated lesion in the anterolateral aspect of the metadiaphyseal region of the lower femur under the cortex, with a cortical defect but no appreciable expansion. Intramedullary extension was not seen. Differential diagnoses at this stage included solitary bone cyst, aneurismal bone cyst, and chondromyxoid fibroma [8]. Due to the rarity of intraosseous schwannoma and its nonspecific clinical and radiological findings, it is very rarely diagnosed preoperatively.

At surgery, a large tumor expanding the lower anterior end of her femur was found. The tumor was partly cellular and partly gelatinous. A regular punched out area where the tumor appeared to have extended out of the femur anteriorly was found. Abnormal-looking tissue extending outside her femur was excised. The defect in her femur was expanded and the whole tumor evacuated. The lower end of her femur (supracondylar area) was severely weakened after resection of the tumor. Prophylactic internal fixation was done with a locking distal femoral plate (Fig. 1d). A bone graft obtained from the iliac crest mixed with bank bone was used to graft the defect.

**Fig. 2:**
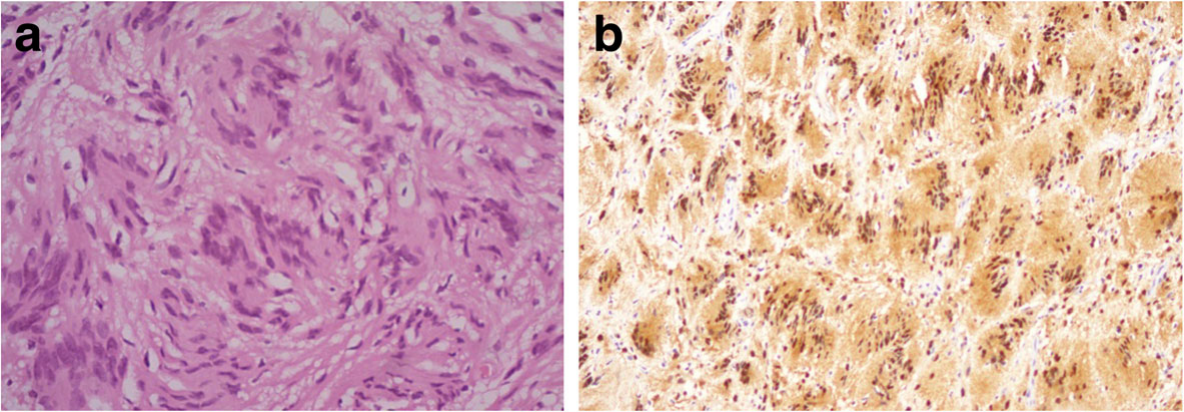
**a** Characteristic microscopy of Antoni A area of schwannoma showing whorled areas and Verocay bodies with nuclear palisading (hematoxylin and eosin × 400). **b** Immunohistochemical staining with S100 shows characteristic nuclear positivity

Hematoxylin and eosin-stained sections revealed a benign spindle cell tumor showing cellular, hyalinized, and hypocellular microcystic areas with areas showing a focally whorled appearance. The spindle cells contained elongated nuclei, characteristic Verocay body-like structures with nuclear palisading (Fig. 2a), and included occasional hyperchromatic bizarre forms. There was no evidence of necrosis or mitotic activity. Immunostaining for S100 was uniformly positive (Fig. 2b). The smooth muscle markers desmin and smooth muscle actin (SMA) were negative. Ki67 showed a proliferation index of <5%.

Microscopy of the tissue removed from outside her femur showed fat necrosis and fibrosis, with no evidence of tumor.

## Discussion

Schwannomas are benign nerve sheath tumors which originate from Schwann cells. They usually occur in soft tissue and are often found in the peripheral nerves of the head and neck or on the extensor extremities. They may be seen at all ages, but occur predominantly in the 20- to 50-year age group. No gender predilection is noted.

They are usually solitary and sporadic, but a few occur in the setting of neurofibromatosis type 2. They are slow growing tumors, which displace nerve fascicles as they expand and are often present for several years before diagnosis. When occurring in bone, they are often asymptomatic, with pain or other neurological manifestation a rare presenting feature [1].

Schwannomas in bone are extremely uncommon, accounting for <0.2% of primary bone tumors. Fewer than 200 have been described in the world literature [2]. Of these, the majority have been described in the skull, including the mandible. Outside the skull, they have been described in the vertebrae and sacrum [3, 9]. The frequency of intraosseous schwannomas in the head and neck has been explained by the high density of sensory nerves in these locations, from which schwannoma are recognized to arise. Of interest, the recognized dearth of such fibers within bone has been thought to account for the rarity of schwannoma in an osseous location [8, 10, 11].

The cause for their relatively common occurrence in the mandible and sacrum have also been discussed. Traditionally, this has been attributed to the presence of long nerve segments which traverse these bones, although this theory remains debatable [4, 12].

Schwannomas are thought to involve bone by one of three mechanisms. An extra-osseous tumor arising from nerves in soft tissue may cause secondary erosion of bone. They may also arise either from nerves entering bone through nutrient canals or within the medullary cavity from non-myelinated nerves associated with blood vessels [3, 8, 10–12].

Schwannoma occurring in the long bones are exceptionally rare. When solitary and not associated with neurofibromatosis, they are thought to arise either from nerves that accompany the nutrient vessels of bone in the diametaphysis or from nerves of the periosteum [2, 10].

Only approximately 20 cases of intraosseous schwannoma involving the long bones have been reported in the literature including tibia [1, 9, 13–15], humerus [2, 16], radius [17], ulna [18], and fibula [1, 19]. Only four cases have been reported in the femur [4–7]. Their clinicoradiologic characteristics are summarized in Table 1 and compared with the current case.

**Table 1:** Patient and tumor characteristics and follow-up of previously reported schwannoma of femur, compared with the current case

Author	Year	Age/Sex	Presenting clinical feature	Site	Size	Radiology of tumor	Recorded follow-up
Sanado *et al*. [7]	1991	17 years M	Pain	Distal femur	>6 cm	Central, osteolytic	NED 1 year
Verma *et al*. [6]	2002	38 years M	Pain	Mid femur	2.2 cm	Eccentric, cortical scalloping	NED 2 years
Hoshi *et al*. [4]	2012	44 years F	Pain	Neck femur	9.1 cm	Eccentric, osteolytic	NED 2 months
Wang *et al*. [5]	2014	42 years M	Incidental radiographic lesion	Distal femur	7.2 cm	Eccentric, osteolytic	NED 5 months
Current case	2016	56 years F	Incidental radiographic lesion, following fall	Distal femur	15 cm	Eccentric, osteolytic	NED 2 years

*F* female, *M* male, *NED* no evidence of disease

Our patient had no evidence of disease at her most recent follow-up, 2.5 years after surgery and had returned to normal activities.

## Conclusions

The purpose of this paper is to call attention to the fact that a common soft tissue tumor like schwannoma can also present as a primary bone tumor. Its nonspecific clinical and radiological findings together with its exceptional rarity of long bone involvement, makes it less likely to be considered in an initial differential diagnosis of lytic lesions of bone. Histological evaluation following biopsy is key to diagnosis.
